# Temporary Absence of Warming in the Northern Weddell Sea Validates Expected Responses of Antarctic Seals to Sea Ice Change

**DOI:** 10.1111/gcb.70290

**Published:** 2025-06-18

**Authors:** M. J. Dunn, C. M. Waluda, S. Adlard, D. Fox, A. S. Lynnes, T. I. Morley, J. Forcada

**Affiliations:** ^1^ British Antarctic Survey Natural Environment Research Council Cambridge UK; ^2^ International Association of Antarctica Tour Operators Providence Rhode Island USA

**Keywords:** Antarctic seals, environmental change, population, sea ice, South Orkney, synchrony

## Abstract

The temporal abundance of Antarctic seals is known to be influenced by variation in the sea ice environment, itself affected by temperature and daylight cycles across seasons. However, the sensitivity of seal populations to changes in their environment beyond expected natural variation depends on their abilities to deal with extreme conditions and to take advantage of favourable environmental changes, which is rarely validated empirically. Here, we report on the responses of three sympatric Antarctic seal species to sea ice change: two ‘ice‐tolerant’ species: Antarctic fur seal *Arctocephalus gazella* and southern elephant seal *Mirounga leonina* , and the ‘ice‐obligate’ Weddell seal *Leptonychotes wedellii*, over a 48‐year period from 1977 to 2024 at Signy Island, South Orkney Islands. This area has undergone changes in the sea ice environment following persistent long‐term warming since the 1950s, and a cooling period from approximately 1998–2014. The shared environment and varying adaptation to sea ice of the three species presented a unique opportunity to assess and compare species‐specific responses to fluctuating sea ice cycles, and validate predictions of responses from projections of sea ice change. Across five decades, all three species were significantly affected by temporary changes in the sea‐ice environment, which contributed to synchronous between‐year variation in numbers, although density dependence was an important effect for each species. Maximum synchrony between seal species was observed from the late 1990s to mid‐2000s, coinciding with the temporary absence of warming, with the ice obligate Weddell seal benefiting from an extended sea ice season but not the two ice‐tolerant species. Numbers of Antarctic fur seals and Weddell seals declined significantly between 1977 and 2024 by approximately 47% and 54% respectively, from a peak in 1994 and 1985, although no significant overall long‐term decline in the numbers of southern elephant seals was found, despite trend synchronicity.

## Graphical abstract

We examine Antarctic seal responses to sea ice change using one of the longest population monitoring records of three seal species: Antarctic fur seal, Weddell seal, and southern elephant seal at Signy Island, in the South Orkney Island group. Across five decades from 1977 to 2024, all were significantly affected by temporary changes in their sea‐ice environment, with Antarctic fur seals and Weddell seals declining significantly by approximately 47% and 54%, respectively. Our study highlights a current and potential future change in the distribution of Antarctic seals and the ongoing impact of climate‐driven habitat change on their populations.

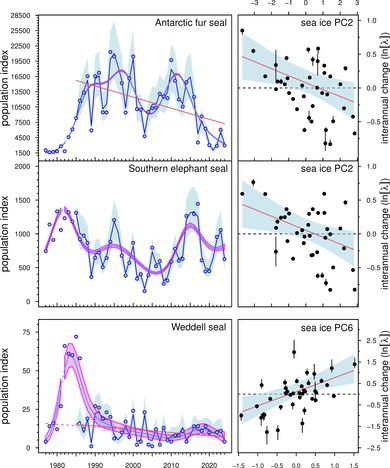

## 1 Introduction

Antarctic seals occupy a circumpolar distribution associated with sea ice, but differences in the life history and adaptations of each species determine their habitat preferences (Gilbert and Erickson 1977; Laws 1984; Siniff 1991). They can be classified into two broad groups, including ‘ice‐obligate’ pack‐ice seals, which rely on sea ice for critical parts of their life history and are sensitive to even small changes in the sea ice environment; and ‘ice‐tolerant’ seals, occurring on or near sea ice but mostly requiring ice‐free areas for reproduction on land (but see Laws 1956). Both groups are expected to be sensitive to sea ice effects on food webs, particularly with increasing industrial fishing (Siniff et al. 2008), but also to changes in sea ice as a substrate.

Rapid environmental change has been taking place across the Western Antarctic Peninsula (WAP) and wider Scotia Arc (SCAR 2009)—including the northern Weddell Sea—involving increases in ocean and air temperatures (Ducklow et al. 2007; Gille 2008; Meredith and King 2005; Turner et al. 2016), and reductions in seasonal sea‐ice duration and extent (Forcada et al. 2006; Stammerjohn, Martinson, Smith, and Iannuzzi 2008; Stammerjohn, Martinson, Smith, Yuan, et al. 2008; Vaughan et al. 2003). The role of sea ice dynamics in influencing population shifts in upper‐trophic predators has been shown to occur directly through changes in habitat availability, and indirectly through the role of sea ice as a mediating link between predator and prey (Morley et al. 2019; Trathan et al. 2007). Since the WAP and Scotia Arc also support large populations of marine mammals and seabirds, together with significant stocks of the Antarctic krill ( *Euphausia superba* ) which is a key species within the regional ecosystem, large‐scale environmental changes may in turn be capable of affecting habitat quality and food availability for a range of Antarctic marine predator species, including marine mammals (Forcada and Hoffman 2014; Forcada et al. 2005; Morley et al. 2019; Silber et al. 2017). Significant population trends in seabird and seal census data across the WAP and Scotia Arc region have previously been linked to environmental indices such as the El Niño‐Southern Oscillation (Croxall et al. 2002; Trathan et al. 2007; Trivelpiece et al. 2011) and the Southern Annual Mode (Forcada and Trathan 2009; Forcada et al. 2008).

Variation in the sea ice environment, which is driven by high seasonality in daylight cycles and temperature (Murphy et al. 2014), is known to influence the temporal abundance of seals depending on species‐specific habitat requirements (e.g., Forcada et al. 2012; Hückstädt et al. 2020; Siniff et al. 2008; Waluda et al. 2010). But how these affect a species' sensitivity to change depends on its tolerance to extreme conditions (Latimer and Zuckerberg 2019; van de Pol et al. 2017), outside the natural variation expected to support survival and reproductive success, and how its populations can benefit from cycles of favourable conditions due to natural variation.

**FIGURE 1:**
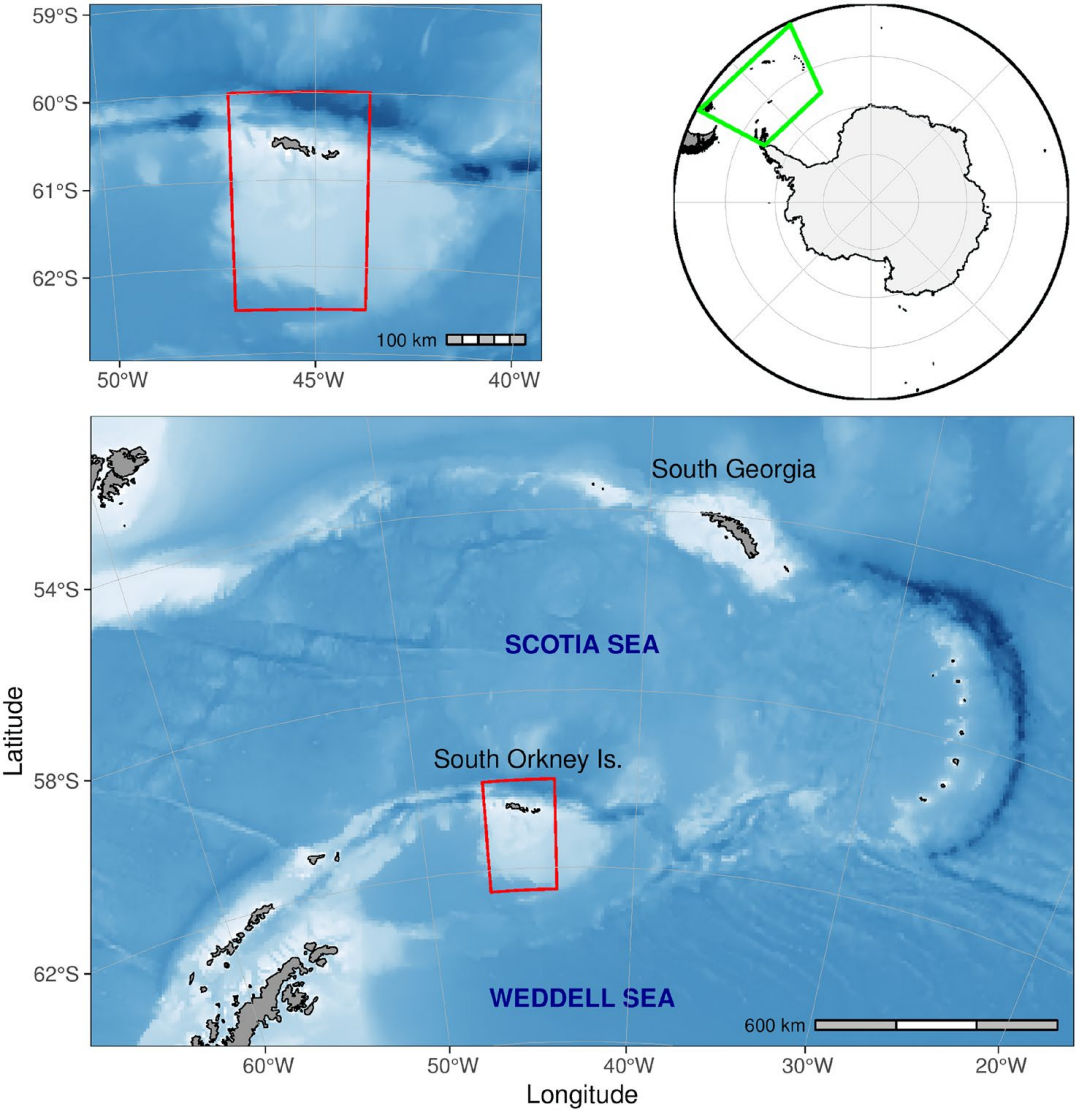
Map of the South Orkney Islands, and of the wider Scotia Sea and northern Weddell Sea, in the southwest Atlantic region of the Southern Ocean. The red polygon delimits the area of satellite derived sea‐ice concentration data used to evaluate long‐term variation in sea‐ice season.

Obtaining accurate population counts at appropriate scales and frequencies is essential to understanding patterns of abundance in wildlife populations (such as seals and seabirds) and their drivers, both at local and regional scales, including in the Antarctic (Bester 2021; Croxall et al. 2012; Forcada 2021). Here, we examine responses to sea ice change using one of the longest population monitoring records of three species with contrasting sea‐ice dependence, the ice‐obligate Weddell seal (*Leptonychotes wedellii*), the ice‐tolerant Antarctic fur seal ( *Arctocephalus gazella* ) and ice‐tolerant southern elephant seal ( *Mirounga leonina* ), collected over 48 years at Signy Island, South Orkney Islands (60°42′ S, 45°36′ W; Figure 1). Despite a persistent long‐term warming since the 1950s across the Antarctic Peninsula and Scotia Arc/northern Weddell Sea (Gorodetskaya et al. 2023; Turner et al. 2020), there was a cooling period from approximately 1998–2014, also known as ‘global warming hiatus’ (Trenberth 2015), which led to mid‐term changes in the sea ice environment around the northern Weddell Sea and Antarctic Peninsula (Turner et al. 2016). This provides a unique opportunity to assess the response of sympatric Antarctic seals to natural variation in a context of sea ice change, and test predictions on future sensitivity, based on previous assessments (e.g., Forcada et al. 2012; Learmonth et al. 2006; Siniff et al. 2008).

Given a shared environment and a contrasting adaptation to sea ice, we anticipated that large natural decadal‐ and sub‐decadal‐scale variability of the region would determine variation in seal abundance through major changes in the sea‐ice season; and hypothesised that sea ice change would temporarily alter interactions between species, which would show similar or alternative environmental responses depending on their tolerance to fluctuating sea ice cycles. We thus investigated the coherence in patterns of count fluctuations across species, the differential contribution of seasonal sea ice cycles to between‐year and longer‐term population responses, and to potential synchrony patterns across species in response to common drivers.

## 2 Methods

**FIGURE 2:**
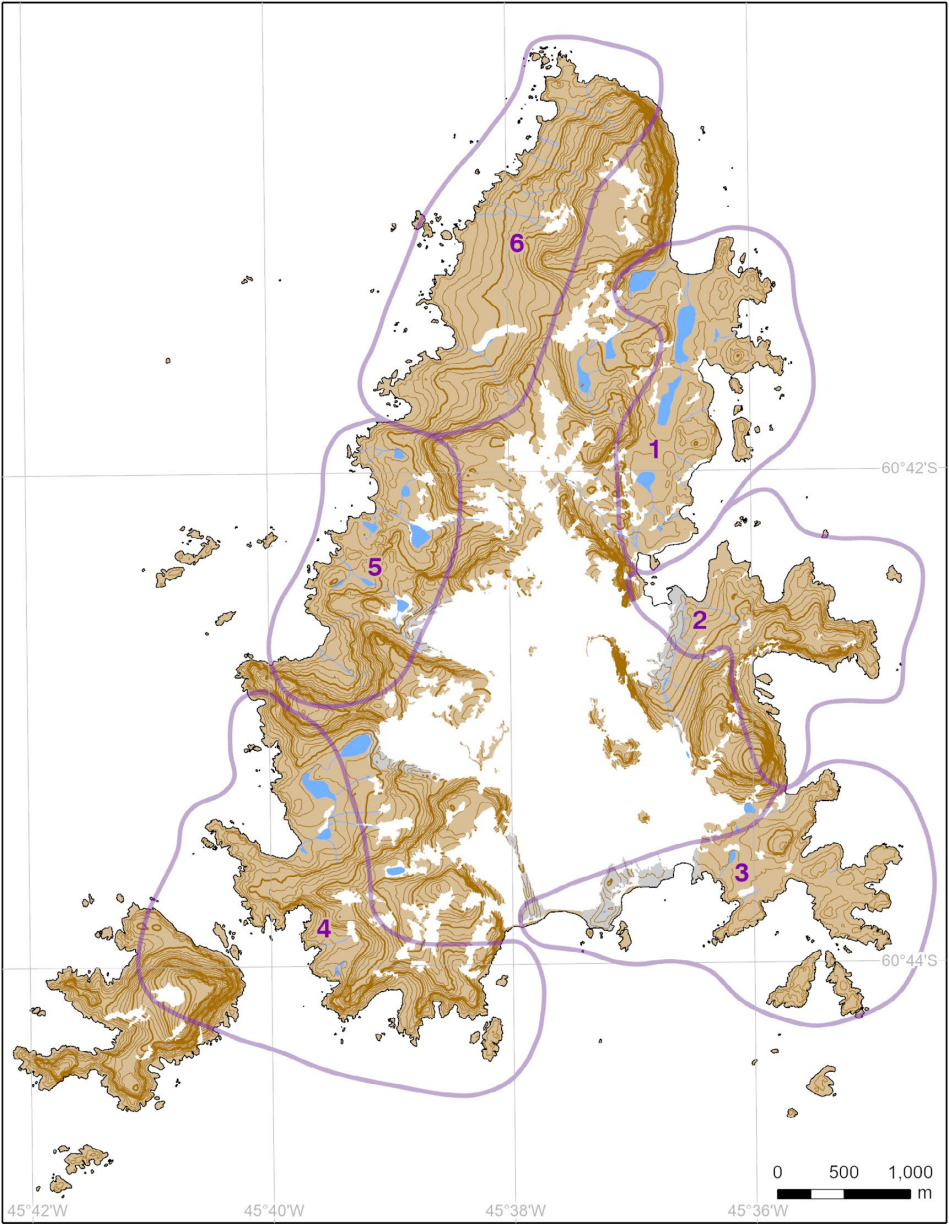
Signy Island, South Orkney Islands, showing the six zones in which the seal surveys took place. Note no seals were located outside of these zones. The blue areas indicate freshwater lakes, white areas permanent ice cover and grey areas unstable moraines. Terrain is indicated by 10 m contours, shown at 50 m intervals.

Between 1977 and 2024, all Antarctic fur seals, southern elephant seals, and Weddell seals present at Signy Island, South Orkney Islands, (Figures 1 and 2) were counted annually as part of a systematic whole‐island seal census (Dunn et al. 2025). Censuses for all three species usually took place between 23 and 25 February, although sometimes as early as 17 February and as late as 4 March, depending on the availability of personnel and weather conditions. Care was taken to carry out the surveys in dry and calm weather conditions, as high levels of precipitation and strong winds are known to drive large numbers of Antarctic fur seals into the sea for the duration of a storm at Signy Island (M.J. Dunn, Pers. Obs). All surveys consisted of direct ground counts led by experienced observers using a consistent methodology. Each year, the coastal areas of the island in which seals are known to haul out were divided up into six separate standard zones with their boundaries unaltered, within which surveys took place (Figure 2). Pairs of observers used tally counters to record each individual seal sighted on land and on sea ice adjacent to the coast of each zone, the whole census being completed over a single day prior to 1995 and over 2 or 3 days from 1997 (no data collected in 1996 and 2021) to 2024, as per the methodology given in Waluda et al. (2010). Most southern elephant seals and Antarctic fur seals were located in favoured haul‐out areas either on or adjacent to the low‐lying beaches of the east and south coasts (Figure 2). Most Weddell seals were sighted on coastal fast ice, this being defined as winter sea ice still held in place during the austral spring and summer months within the various coves, bays, and inlets at Signy Island.

### 2.1 Sea Ice Variables

We used satellite measurements of percent sea ice concentration (data available from 1982 onwards) from the NOAA/NESDIS/NCEI Daily Optimum Interpolation Sea Surface Temperature (SST), version 2.1, dataset (DOISST v2.1) (Huang et al. 2021), to determine inter‐annual variation in the ice edge, as estimated by the 15% sea ice concentration threshold. We selected data with a resolution of 1/4° daily at a polygon (Figure 1) of longitude range [−44°, −47°], latitude range [−60°, −62°], and total area of 37,966km^2^, with the South Orkney Islands at approximately −46.875° and −61.875° (dataset ‘ncdcOisst21Agg_LonPM180’; URL: https://coastwatch.pfeg.noaa.gov/erddap/). Sea ice concentration (from 0% to 100%) was the relative amount of a grid cell of 1/4° covered by ice, and for a larger area it was the sum of the area of each cell multiplied by the fractional concentration for that cell. Sea ice extent defined a region as either ice‐covered or uncovered as a binary term; a grid cell had ice (1) or no ice (0), as defined by the 15% concentration threshold.

For each year from 1982 to 2024, when sea ice records were available, we considered an annual period starting approximately around the date of mean sea ice extent minimum on February 16; i.e., day 46 in year 1 to day 410 or 411 in leap years‐of year 2. We then calculated the timing of the seasonal sea ice cycle (e.g., Stammerjohn, Martinson, Smith, and Iannuzzi 2008), and annual sea ice extent and area. The derived covariates were: (1) day of advance: first day when sea ice concentration exceeded 15% for at least five consecutive days; (2) day of retreat: first day when sea ice concentration remained < 15% until the end of the period; (3) ice season duration: total number of sea‐ice days, between day of advance and retreat; (4) sea‐ice persistence: percent time sea ice was present between day of advance and retreat; (5) day of minimum sea ice extent; (6) day of maximum extent; (7) day of minimum sea ice area; (8) day of maximum sea ice area; (9) minimum area of the ocean in km^2^ with at least 15% sea ice concentration; and (10) maximum area of the ocean with at least 15% sea ice concentration (Forcada et al. 2025). If the sea ice concentration never decreased below this threshold, the days of advance and retreat were set to the lower and upper limits, respectively. The difference between variables 3 and 4 was due to oscillatory advances and subsequent retreats of the ice edge during autumn or spring, or due to pack ice openings caused by winds and currents.

## 3 Analysis

### 3.1 Sea Ice Principal Components

We expected correlations among sea ice covariates, as these were derived from a single sea ice concentration data set. Thus, we used a principal component analysis (PCA) using the *R* package *FactoMineR* (Lê et al. 2008) to obtain uncorrelated synthetic new variables (PCs = principal components). Using PCs as covariates, we reduced bias from high dimensionality and multicollinearity (Gimenez and Barbraud 2017; Grosbois et al. 2008) and increased the power to detect significant effects. With 10 covariates, we considered the first six PCs, which accounted for 95% of cumulative explained variance, and by comparing different combinations of PCs, we avoided selection of PCs only based on highest explained variation alone (Aguilera et al. 2006; Gimenez and Barbraud 2017).

### 3.2 Population Dynamics, Sea Ice Effects and Synchrony

We used log‐linear models with a discrete‐time stochastic Gompertz formulation (e.g., Dennis and Taper 1994; Lebreton and Gimenez 2013) of the observed count $y_{s,t}$ for species *s* and year *t*, in response to sea ice covariate ($z_{t}$) with fixed effects ($\gamma_{s,t}$). Species interactions were based on a year‐lagged count, $y_{s,t-1}$, and were represented by coefficients $\nu_{s,r}$ for species *s* and *r*, where density‐dependence occurred when *s* = *r*. To evaluate synchrony, we used two independent random effects (e.g., Grosbois et al. 2009; Lahoz‐Monfort et al. 2011; Santin‐Janin et al. 2014). One was a time‐specific random effect, $\delta_{t}\sim N\left(0,\sigma_{\delta }^{2}\right)$ that modelled common synchronous variation across species. A separate time‐specific random effect for each species *s*, $\varepsilon_{t,s}\sim N\left(0,\sigma_{\varepsilon }^{2}\right)$, accounted for additional, asynchronous variation.

We used a negative binomial error distribution (e.g., Davis and Wu 2009) to accommodate zero inflation and positive skewness from low counts. For species *s* at time *t*,

$$y_{s,t}\sim \mathrm{NB}\left(r_{s},p_{s,t}\right)$$

where $r_{s}$ was the dispersion parameter for species *s*, and $p_{s,t}$ was the success rate parameter,

$$p_{s,t}=r_{s}/\left(r_{s}+E\left[y_{s,t}\right]\right)$$

For single species, $\log E\left[y_{t}\right]=x_{t}$ and $E\left[y_{t}\right]$ was modelled as

(1) $$\log\left(\frac{x_{t}}{x_{t-1}}\right)=\alpha +\left(1-\nu \right)y_{t-1}^{T}+z_{t}^{T}\gamma_{s}+\varepsilon$$

where α is an intercept, or intrinsic growth rate and equivalent to $\log\left(\lambda \right)$ when $E\left[y\right]=1$, ν is a density‐dependence coefficient, and ε and error term.

For multi‐species synchrony models, $\log E\left[y_{s,t}\right]=x_{s,t}$, $E\left[y_{s,t}\right]$ was modelled as

(2) $$\log\left(\frac{x_{s,t}}{x_{s,t-1}}\right)=\alpha_{s}+\sum_{j=1}^{n_{s}}\nu_{j,s}y_{j,t-1}^{T}+z_{t}^{T}\gamma_{s}+\delta_{t}+\varepsilon_{t,s}$$

where $\alpha_{s}$ is a species‐level random effect with distribution $\alpha_{s}\sim \text{Normal}\left(\bar{\alpha },\sigma_{\alpha }^{2}\right)$.

The sum of the other random terms' variances, ${\sigma_{\delta }^{2}+\sigma }_{\varepsilon_{s}}^{2}$, was between‐year variance unexplained by sea ice covariates and species interactions. These variances were identically partitioned among species in a shared component $\sigma_{\delta }^{2}$, but not among unshared components $\sigma_{\varepsilon_{s}}^{2}$. The fraction of between‐year variance accounted by the shared component (Grosbois et al. 2009; Lahoz‐Monfort et al. 2011; Santin‐Janin et al. 2014), equivalent to the intra‐class correlation ${\mathrm{ICC}}_{S}=\sigma_{\delta }^{2}/\left({\sigma_{\delta }^{2}+\sigma }_{\varepsilon_{s}}^{2}\right)$, was a measure of the synchrony of species *s* with the rest of the species. ${\mathrm{ICC}}_{S}$ tended to one when the shared (synchrony) component was large relative to the unshared component and tended to zero otherwise.

### 3.3 Contribution of Sea‐Ice Covariates to Synchrony

The contribution of sea‐ice covariates to between‐species synchronous $\delta_{t}$ and asynchronous $\varepsilon_{t,s}$ components were obtained following Grosbois et al. (2009) and Lahoz‐Monfort et al. (2011) as

$$C_{\delta }=1-\frac{{\hat{\sigma }}_{\delta }^{2}\left(\mathrm{res}\right)}{{\hat{\sigma }}_{\delta }^{2}\left(\text{total}\right)}$$

$$C_{\epsilon_{s}}=1-\frac{{\hat{\sigma }}_{\epsilon_{s}}^{2}\left(\mathrm{res}\right)}{{\hat{\sigma }}_{\epsilon_{s}}^{2}\left(\text{total}\right)}$$

where estimates of ${\hat{\sigma }}_{\delta }^{2}$ and ${\hat{\sigma }}_{\epsilon_{s}}^{2}$ were derived from a model incorporating total between‐year variance (total), and a model with residual variance (res) which included sea‐ice effects.

### 3.4 Population Model Fitting, Selection, and Assessment

We fitted population and subsequent Bayesian models with Markov Chain Monte Carlo (MCMC) methods in *BUGS* language and program *JAGS* (Plummer 2003), run from *R* (v4.4.1; R Core Team 2024) with package *rjags* (Plummer 2023) and its wrapper *jagsUI* (Kellner 2021). To investigate sea ice effects and species interactions with synchrony, we had 32,768 possible model combinations of nine $\nu_{s,r}$ and six $\gamma_{s}$ parameters (Equation 2). Thus, we used indicator‐based model selection (Kuo and Mallick 1998; O'Hara and Sillanpää 2009) to identify variables with high explanatory power. For species interactions ($\nu_{s,r}$; and similarly for sea ice effects, $\gamma_{s,t}$) we set $\theta_{s,r}=I_{s,r}\nu_{s,r}$, where $\nu_{s,r}$ was selected when $I_{s,r}$ =1, and set to 0 for $I_{s,r}$ =0. We used the following prior distributions: $\nu_{s,r}\sim N\left(0,\sigma_{\nu }^{2}\right)$, with $\sigma_{\nu }^{2}\sim \Gamma^{-1}\left(1,0.001\right)$, and $\gamma_{k}\sim N\left(0,\sigma_{\gamma }^{2}\right)$, with $\sigma_{\gamma }^{2}\sim \Gamma^{-1}\left(1,0.001\right)$; and a prior for the indicator distribution, $I_{s,r}\sim \text{Bernoulli}\left(\iota \right)$, with $\iota \sim \text{Beta}\left(2,8\right)$. We fitted models of synchrony with priors $\bar{\alpha }\sim N\left(0,10^{-2}\right)$, $\sigma_{\alpha }\sim U\left(0,2\right),$ $\sigma_{\varepsilon_{s}}^{2}\sim \Gamma^{-1}\left(1,0.001\right)$, for each *s*, and $\sigma_{\delta }^{2}\sim \Gamma^{-1}\left(1,0.001\right)$, and $r_{s}\sim U\left(0,50\right).$

First, we investigated species interactions in a multi‐species synchrony model without ice effects (Equation 2; Table S2). Biologically meaningful interactions were only expected among certain combinations of species (e.g., fur seals and elephant seals, but less so on Weddell seals and fur seals, which are more segregated in habitat). After inspection of the data, we retained models with interaction terms that mostly excluded 0 from the 95% credible interval and had coefficients of interest with a proportion equal to or above 96% of the posterior distribution with the same sign as the mean, all of which supported a significant effect. Next, we fitted single‐species population models including density dependence to investigate best combinations of sea‐ice effects by species (Equation 1; Table S3). And finally, because the main interest was identifying different species with shared or unshared responses to sea ice (e.g., same or different regression coefficients across species), we used WAIC‐based model selection (Gelman et al. 2014; Watanabe 2010) only in a reduced model set based on covariates with higher indicator probabilities (Equation 2; Table S4).

In all Bayesian analyses, we used 200,000 iterations of four Markov chains and discarded the first 100,000 samples of each chain as burn‐in phase, thinning the remainder to every 100th sample, which produced 4000 posterior distribution samples. We assessed chain convergence visually using trace plots, through the mixing of the chains and sample autocorrelation plots, and using the $\hat{R}$ potential scale reduction factor statistic of less than 1.05 to retain posterior distribution samples (Vehtari et al. 2021). We assessed the ability of the models to generate data that were consistent with the observed data using posterior predictive checks (Gelman et al. 2000).

### 3.5 Population Variation and Trends

To examine non‐linear trends and long‐term variation in annual counts for each species we fitted Bayesian generalised additive models (GAMs) with a Poisson error structure. The mean annual count ($y_{t}$) for season *t* = 1, …, *T* was $y_{t}\sim \text{Poisson}\left(\mu_{t}\right)$, with $\log\left(\mu_{t}\right)=\beta_{0}+\sum_{k}b_{k}\left(T_{t}\right)\beta_{k}$, where $T_{t}$ is the survey season, *b*_*k*_ the basis of a penalised regression spline. We selected multivariate Normal smoothing penalty matrices using R package *mgcv* (v.1.9‐0; Wood 2016) to specify priors for multivariate normal precision matrices. These were directly incorporated into JAGS models to obtain estimates of $\mu_{t}$ for prediction.

We assessed interannual population change from estimates of $\log\left(\lambda_{s}\right)$ and geometric mean $\lambda_{s}$ derived from estimates of $E\left[\hat{y_{s,t}}\right]$ of multi‐species models (Equation 2). We compared these estimates from those obtained with linear models of log‐transformed mean counts ($x_{t}$ = $\ln(y_{t}$)) against season, using robust‐resistant regression MM‐method models (Yohai 1987). When regression slopes were found to be significantly different from zero, we assessed long‐term and annual declines for the affected species. Precision was estimated using a parametric bootstrap of the linear model residuals (Davison and Hinkley 1997).

### 3.6 Additional Measures of Synchrony

As there are different definitions of synchrony with different assumptions and restrictions as metrics of species associations, we evaluated different alternatives. These included Kendall's coefficient of concordance (*W*) (Buonaccorsi et al. 2001; Legendre 2005), the mean Pearson's correlation coefficient ($\bar{\rho }$) (Houlahan et al. 2007; Purves and Law 2002), and the community dynamics (*φ*) of Loreau and de Mazancourt (2008), all of which range between 0, for no synchrony, and 1, for maximum synchrony. For this we used the implementations in *R* package *synchrony* (Gouhier and Guichard 2014), and obtained 4999 simulations in permutation tests to assess the level of significance (*p* value). We obtained synchrony metrics for temporal species counts ($y_{s,t}$) and for interannual changes in species counts, $x_{t}-x_{t}$_−1_, where $x_{t}$ = $\ln(y_{t}$).

### 3.7 Scales and Patterns of Synchrony in Seal Counts

To identify common scales and patterns in population fluctuations across species, we analysed count variation and how it evolved over time through wavelet decomposition. This highlighted short‐lived (transient) population dynamics, which combined led to mean synchrony. We used wavelet power analyses $W_{x}\left(f,\tau \right)$ for counts of single species (*x*), and wavelet coherence of pairs of time series ${Ρ}_{x,y}\left(f,\tau \right)$, using *R* package *biwavelet* (v0.20.21; Gouhier et al. 2021). Wavelet coherence provided a direct measure of the correlation between the power spectra of two species (x, y), or a species and a sea‐ice effect, revealing common patterns of gradual change (Cazelles et al. 2008; Torrence and Compo 1998). For the three species together, we used a multivariate coherence analysis based on a localised wavelet modulus ratio $\rho \left(t,s\right)$ as implemented in *R* package *mvcwt* (Keitt 2008, 2014). A coherence value of 1 meant a linear relationship (synchrony) between two or more species around a certain year on a scale of *s* years, whereas a 0 meant no correlation. We used phase differences to understand the delay or synchronisation between oscillations of two time series.

With a time‐series length of 48 years, low‐frequency components with scales greater than 8–9 years, corresponding to approximately one fifth of the total length, could not be well resolved and were removed with an 8‐year high‐pass Gaussian filter (Jenouvrier et al. 2005; Park and Gambéroni 1995). The filtered time‐series were also normalised to have mean 0 and SD 1, and our results focused on scales of between 2 and 8 years.

## 4 Results

### 4.1 Sea Ice Environment

The first two principal components together explained over 60% of the variance (Table S1; Figure S1a,b), describing potential variation in changing seasonal sea ice cycles over a 40‐year study period (1985–2024; available satellite record for sea‐ice when count data were available without major interruptions). PC1 reflected an increasing sea ice season duration, day of ice retreat, and minimum and maximum area covered by ice. It was highly negatively correlated with advance day and days of maximum and minimum extent (Table S1; Figure S1a). PC2 was significantly correlated with all variables except maximum area and day of minimum area. The highest correlations were with retreat day and day of maximum area, followed by days of maximum and minimum extent and minimum sea ice area (Table S1; Figure S1a).

### 4.2 Seal Population Dynamics and Trends

All models had an $\hat{R}$ convergence diagnostic less than or equal to 1.05. Posterior predictive checks confirmed that the fitted multi‐species synchrony model could generate data without obvious systematic discrepancies with the observed data, i.e., 39% of counts simulated from the fitted model had lower root mean square error (RMSE) than the observed counts (Bayesian *p*‐value = 0.39; values close to 0.5 indicate a reasonable fit).

**FIGURE 3:**
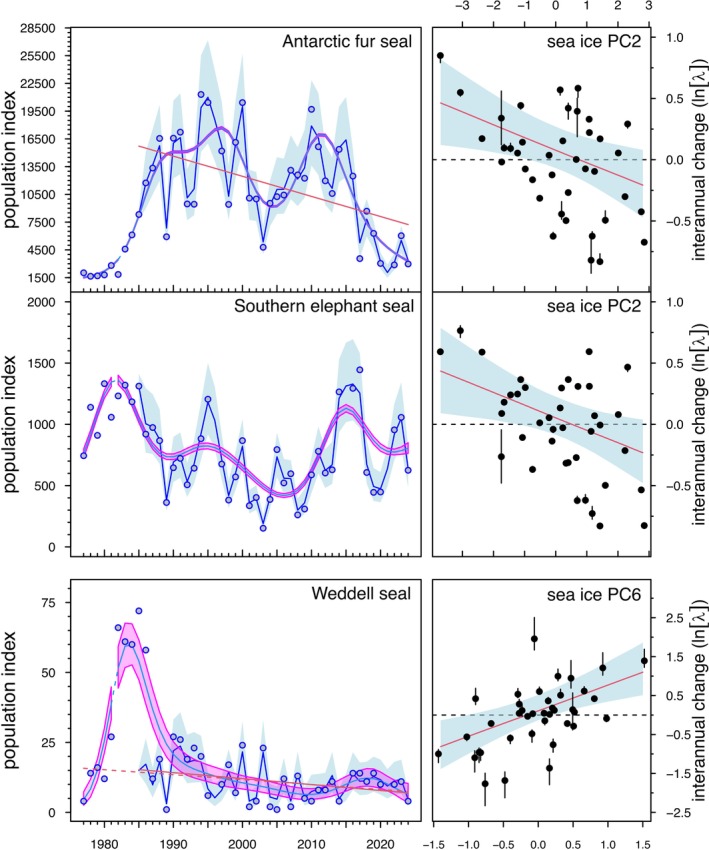
Population model predictions and best modelled sea‐ice effects for Antarctic fur seals, southern elephant seals and Weddell seals at Signy Island, South Orkney Islands, from 1977 to 2024. Left panels show mean counts with blue polygons showing 95% credible intervals for fitted multi‐species population dynamics models from 1985. Magenta polygons represent the pointwise 95% credible‐interval limits for fitted Poisson GAMs for the entire count series. Dashed lines indicate periods without data, and red lines are fitted robust‐resistant linear regression trend models. Right panels show population growth rate (log‐lambda) against sea ice principal components according to best modelling options. Black dots are predicted values with 95% credible intervals in vertical bars.

Model selection and parameter estimates did not support significant and meaningful inter‐species interactions but retained density‐dependent effects in all species (Tables S2 and S3). From the best synchrony model (model 6; Table S4), the geometric mean lambda for Antarctic fur seals was estimated as 0.984 [0.974, 0.995], equivalent to an annual decline of 2% since 1985; and for southern elephant seals, the geometric mean lambda was estimated as 0.993 [0.985, 1.001], equivalent to an almost significant annual decline of 1%. For Weddell seals, the estimate was 0.987 [0.974,1.000], equivalent to an annual decline above 1%. These results were consistent with long‐term decline estimates obtained with robust‐resistant‐linear regression models (Table 1; Figure 3).

**TABLE 1:** Results of the population dynamics models and robust regression analyses of seal species counts at Signy Island to examine trends. For $\ln\left(\lambda \right)$, values between squared brackets are 95% credible intervals from posterior distribution simulations. For other parameters, these are 95% confidence intervals, standard errors in parentheses, and NS is non‐significantly different from 0.

Species	$\ln\left(\lambda \right)$	Regression slope (log‐scale)	Long‐term decline 1977–2024 (%)	Annual decline (%)
Antarctic fur seal	0.984 [0.974, 0.995]	−0.020 (0.008)	−46.7 [−10.3, −74.4]	−1.8 [−3.5, −0.3]
Elephant seal	0.993 [0.985, 1.001]	−0.0001 (0.0074)	NS	NS
Weddell seal	0.987 [0.974,1.000]	−0.027 (0.013)	−53.7 [−5.4, −88.2]	−2.0 [−4.8, −0.1]

The Poisson GAMs showed a high temporal variation in counts of each species. Antarctic fur seals had an initial phase of rapid increase followed by an abrupt decline since approximately 2015, which was consistent with the expansion and subsequent decline of the species at South Georgia (Forcada et al. 2023). The GAMs also showed a period of decline from the late 1990s to approximately 2005 for elephant seals and fur seals, but of slight increase for Weddell seals. This period was characterised by absence of warming and the onset of longer sea ice seasons in the Southwest Atlantic region.

### 4.3 Seal Population Synchrony

Predicted trends in counts based on GAM smooths showed comparable peaks and troughs among species (Figure 3). Accordingly, there were significant synchrony values in $y_{t}$ among calculation methods (Table 2), except for $\bar{\rho }$, which is a less reliable metric when the number of species is low (Gouhier and Guichard 2014). The synchrony in interannual change in counts ($x_{t}-x_{t}$_−1_) was consistently high across species and between species pairs. Therefore, there was support for an association of seal counts with long‐term trends, and with inter‐annual variation in external drivers.

**TABLE 2:** Temporal synchrony measures using mean correlation coefficient ($\bar{\rho }$), Kendall's coefficient of concordance *W*, and community dynamics *φ* for seal counts ($y_{s,t}$), and interannual change in counts, $x_{s,t}$−$x_{s,t}$_−1_ where $x_{s,t}$ = $\ln\left(y_{s,t}\right)$, for each species *s* and between species pairs.

Metric	All species		AFS‐SES		AFS‐WDS		SES‐WDS	
	$y_{t}$	$x_{t}$‐$x_{t}$_−1_	$y_{t}$	$x_{t}$‐$x_{t}$_−1_	$y_{t}$	$x_{t}$‐$x_{t}$_−1_	$y_{t}$	$x_{t}$‐$x_{t}$_−1_
$\bar{\rho }$	0.16 (0.085)	0.75 (< 0.001)	0.25 (0.131)	0.83 (< 0.001)	−0.02 (0.898)	0.68 (< 0.001)	0.26 (0.114)	0.73 (< 0.001)
W	0.44 (0.048)	0.77 (< 0.001)	0.65 (0.033)	0.90 (< 0.001)	0.49 (0.578)	0.79 (< 0.001)	0.61 (0.094)	0.80 (< 0.001)
ϕ	0.91 (0.067)	0.83 (< 0.001)	0.92 (0.065)	0.91 (< 0.001)	0.99 (0.560)	0.84 (< 0.001)	0.94 (0.060)	0.87 (< 0.001)

*Note:* *p*‐values, in parentheses, are from significant tests based on 4999 permutations.

Abbreviations: AFS, Antarctic fur seals, SES, southern elephant seals; WDS, Weddell seals.

The *ICC* statistics from multi‐species population models (Equation 2) suggested a synchrony among species of 0.404 [0.163, 0.671]. The synchrony of Antarctic fur seals with the other two species and southern elephant seals with the other two species was 0.406 [0.160, 0.701] and 0.441 [0.187, 0.718], respectively, whereas the synchrony of Weddell seals with the other two species was 0.221 [0.071, 0.506] (Table S5).

### 4.4 Sea Ice Effects on Seal Populations and Contributions to Synchrony

For Antarctic fur seals and southern elephant seals, the sea ice model with highest probability included an effect of PC2 (Table S3). This was best modelled as a shared effect in synchrony models (Table S4), with an estimate of $\gamma_{A}^{\mathrm{PC}2}$= −0.103 [95% credible interval: −0.182, −0.024] (Table S5; Figure 3). For Weddell seals, the sea ice effect with highest probability was of PC6 (Tables S3 and S4), with an estimate of $\gamma_{\mathrm{PC}2}^{W}$ =0.662 [0.275, 1.052] and predicted response shown in Figure 3.

The dynamics of Antarctic fur seal and southern elephant seal populations were negatively related to an earlier day of advance and later day of sea ice retreat, earlier reach of the maximum ice area and extent, and a higher persistence and duration of the sea ice season. For Weddell seals, positive sea ice effects indicate a higher population with later sea ice retreat and an earlier reach of the maximum sea ice area (Figure 3).

The contribution of sea ice effects to the synchronous variation among species was $C_{\delta }=$ 0.074. By contrast, in Weddell seals ($C_{\epsilon_{W}}=$ 0.277) and to a much lower extent Antarctic fur seals ($C_{\epsilon_{A}}=$ 0.109), a higher proportion of between‐year variation asynchronous to the other species was related to sea ice. This proportion was much lower in southern elephant seals ($C_{\epsilon_{S}}=$ 0.049; Table S5), which indicates that while sea ice effects were significant, density‐dependence and other unaccounted sources of between‐year variation were important for all species (see effects in Table S5).

### 4.5 Scales and Patterns of Synchrony in Seal Counts

**FIGURE 4:**
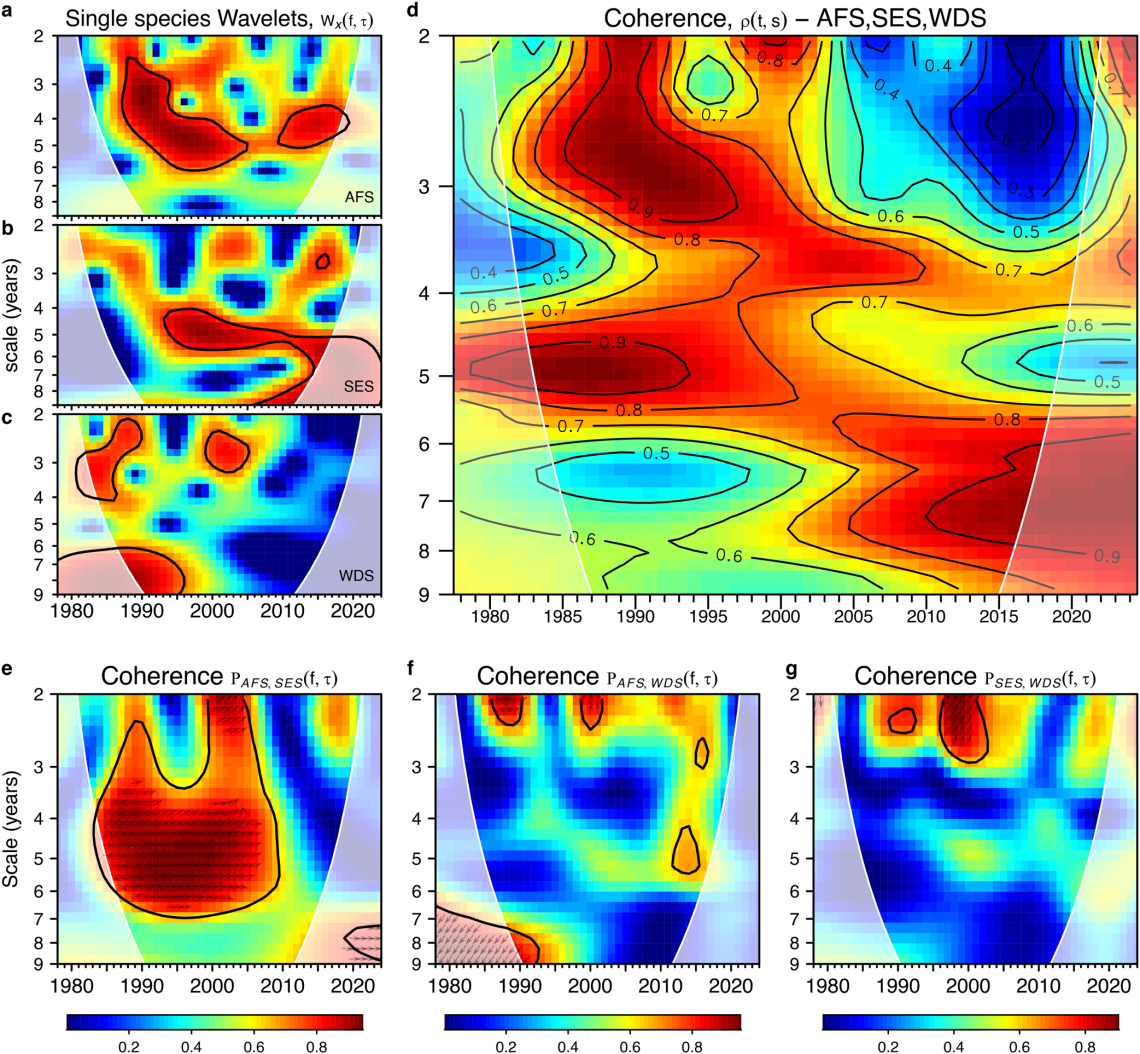
Species wavelet and between‐species coherence wavelet power spectra. In wavelets, warm and cool colours representing high and low power, and in coherence wavelets, these represent areas of correlation between 0 and 1. Multi‐species coherence is measured as the ratio of aggregated species count variation at time *t* and scale *s* to individual species count variation. With synchrony, aggregate variation approaches the sum of individual species variation, and the ratio tends towards one. In all plots, regions of significantly high temporal variation compared to a null model (red noise) are designated with black contours, and paler areas delimit the cone of influence, with outside values being less reliable due to edge effects. Small arrows show phase differences between species *x* and *y*. With right pointing arrows *x* and *y* are in phase; to the left, *x* and *y* are in anti‐phase; pointing up, *y* leads *x* by *π*/2; and pointing down, *x* leads *y* by *π*/2.

The cycles of variation in Antarctic fur seals increased significantly in scale from the mid‐1980s to the mid‐2000s, with initial modes from 2–3 to 5–6 years, which changed to 4–5 years after 2010 (Figure 4a). In contrast, southern elephant seals showed a persistent mode of variation of approximately 5 years since the mid‐1990s (Figure 4b). Together, both species showed maximum coherence in variation between the mid‐1980s and 2010, being in phase, and with an initial scale of 3–6 years which subsequently extended to 2–6 years in the mid‐2000s (Figure 4e). This suggested a maximum synchrony during a period of extended sea ice season.

In contrast, Weddell seals had two significant periods of higher variation at 2–3 years in the late 1980s and early 2000s (Figure 4c). Their maximum coherence in variation with Antarctic fur seals and southern elephant seals was approximately at the same scale and year intervals, with Weddell seals leading the other two species in the early 2000s (Figure 4f,g). This common mode of variation held for the coherence of the three species together (Figure 4d), which also included a significant 5‐year cycle between the late 1980s and mid‐1990s.

The patterns of coherence between seal counts and sea ice PCs are shown in Figure S2. Considering the main significant PC effects for each species, the maximum correlations between seal population and sea ice variation were observed between the late 1990s and early to mid‐2000s (Figure S2d,e), at a scale of 2–3 years for fur seals and elephant seals, and similarly for Weddell seals, which had a larger coherence cycle (from 1995 to 2012) at a low scale, and also a cycle at 4–5 years from 2000 to 2014, approximately (Figure S2r). The window of variation common to all species corresponds with the onset of cooling and increasing sea ice season.

## 5 Discussion

Our results show profound variation in numbers of three Antarctic seal species over nearly five decades, significantly affected by temporary changes in the sea‐ice environment, which contributed to their synchronous between‐year variation. Specifically, seal numbers in February and March were related to earlier annual sea ice advance and later retreat dates, earlier reach of maximum ice area and extent, and higher persistence of and duration of the annual sea ice seasons that preceded these counts. Density dependence was also an important effect on each species, but other large unquantified sources of mid‐ to long‐term variation such as major climactic and oceanographic drivers contributed substantially to population differences and trends. The total numbers of Antarctic fur seals and Weddell seals present at Signy Island declined significantly over the study period, by approximately 47% and 54% respectively. This marks a departure from a previous study of Antarctic fur seal population trends at Signy Island which found a 10‐fold increase in abundance followed by an apparent stabilising of numbers between 1977 and 2008 (Waluda et al. 2010). This study corroborates these earlier findings and indicates that from approximately 2015 the population trend changed profoundly, with a significant decline up to the present (2024).

All counts took place during annual seal moult periods, and it should be recognised that seals are not necessarily as faithful to moulting sites as they are to sites at which they breed: non‐breeding animals disperse to find better feeding habitats and to rest and moult. However, southern elephant seals and Weddell seals do breed at Signy Island and remain for their moult, and all three species use Signy, albeit temporarily, in different ways. Although the modelled numbers of seals for each species do not represent actual population totals but rather moult totals, the modelled trends represent seal preferences, given the variation in the sea ice environment. The decline in Antarctic fur seal numbers at Signy Island (themselves originating largely from the South Georgia breeding population, Boyd et al. 1998) is commensurate with an on‐going decline in the numbers of breeding female Antarctic fur seals at Bird Island, South Georgia (Forcada et al. 2023). However, the actual numbers of seals recorded at Signy Island—for all three species—should be interpreted with caution in the context of the variable environment and restricted regional counting range and treated only in that context. Previous studies have shown similar trends in numbers of Antarctic fur seals at Signy Island and nearby Laurie Island (Waluda et al. 2010), suggesting our data are likely to reflect trends across the South Orkney archipelago, given the close proximity of the neighbouring islands and shared environmental conditions. We found a non‐significant long‐term decline in the numbers of southern elephant seals recorded over the study period, although the species shared a similar period of decline with Antarctic fur seals from the late 1990s to approximately 2005. Indeed, our results indicate that the period of maximum synchrony between seal species was observed from the late 1990s to mid‐2000s and coincided with the temporary absence of warming, which benefitted the ice obligate Weddell seal but not the ice‐tolerant Antarctic fur seal and southern elephant seal. Low to mid‐term changes in sea ice season largely occurred during a cooling phase between the late 1990s and 2015–2016. Since the early 2000s, the maximum sea ice extent and maximum ice area were reached earlier, and the maximum and minimum areas covered with ice increased, together with the duration of the ice season and with a decreasing day of advance and increasing day of retreat. These changes were somehow reversed since 2015, with the resumption of warming in the region (Gorodetskaya et al. 2023; Turner et al. 2020). For Weddell seals, factors affecting access to breeding locations and those affecting sea ice persistence, particularly of the fast ice near breeding locations, have been identified as main factors increasing sensitivity to change (Siniff et al. 2008). At Signy Island, the species was expected to breed more in ‘good’ ice seasons but be more dispersed otherwise (Croxall and Hiby 1983). Our results support that Weddell seals benefitted from an extended sea ice season.

At the South Orkney Islands, the highest counts of Antarctic fur seals are obtained from early January to late February, after the breeding season, and are largely composed of mostly non‐resident juvenile and adult males, of ages 2–7 and older (Laws 1973; Smith 1988). The numbers of breeding females and pups are extremely low, and at Signy Island in particular, where frequent counts have been made, they have declined to almost zero since 2000 (Waluda et al. 2010). The sex and age composition of the population of Antarctic fur seals at Signy Island reflects their lack of temporal or spatial constraints from late December onwards, allowing them to migrate to higher latitudes, as opposed to the spatially restricted females who are limited to localised foraging during austral spring and summer pup rearing (Forcada and Staniland 2009; Jones et al. 2020; Staniland and Robinson 2008; Waluda et al. 2010). Apart from local sea ice effects, the numbers were expected to fluctuate annually with the post‐breeding temporary migration of the largest source populations, notably South Georgia (Boyd et al. 1998; Laws 1973), and over time, reflected the long‐term population trends and cycles at this location (Forcada et al. 2023).

For the ice‐obligate Weddell seals, warming in the Antarctic Peninsula and Scotia Arc/northern Weddell Sea, with accompanying reductions of sea ice leading to reduced critical habitat for resting and breeding, is predicted to have a significant negative effect on their populations (Costa et al. 2010; Forcada et al. 2012). Conversely, for Antarctic fur seals and southern elephant seals in the same region, such reductions in sea ice and retreat of glaciers have led to the areas for moulting, pupping, and breeding increasing: access to beach areas, in turn likely facilitating future population expansion southward (Costa et al. 2010; Siniff et al. 2008). However, future changes in prey availability are a potentially confounding factor in predicting population trends for all three seal species (Negrete et al. 2022; Siniff et al. 2008): in particular, declines in Antarctic krill biomass have been associated with rapid regional warming and subsequent sea ice reduction (Atkinson et al. 2019, 2004; Forcada et al. 2012; McBride et al. 2021). Long‐term studies of Antarctic fur seal population size and breeding success at South Georgia have shown an inverse relationship between sea surface temperatures and breeding success, with increased environmental variability, driven by increasing frequency of positive temperature anomalies, resulting in limited local availability of Antarctic krill both at the South Orkneys (Casaux et al. 2003, 2016) and South Georgia (Cleary et al. 2019; Forcada and Hoffman 2014). Limitations in krill availability have resulted in a consequential loss of life history buffering, through increased fitness costs, for breeding Antarctic fur seals at South Georgia for whom Antarctic krill is a dietary staple (Forcada et al. 2008). Consequently, predicting how future changes in the Antarctic food webs from loss of sea ice and increasing industrial fishing will affect seal populations, including in the South Orkney Islands, especially through negative consequences on Antarctic krill abundance, remains uncertain.

## Author Contributions

**M. J. Dunn:** conceptualization, data curation, formal analysis, funding acquisition, investigation, methodology, project administration, resources, visualization, writing – original draft, writing – review and editing. **C. M. Waluda:** data curation, investigation, methodology, resources, validation, writing – original draft, writing – review and editing. **S. Adlard:** data curation, investigation, methodology, resources, writing – review and editing. **D. Fox:** data curation, investigation, methodology, resources, writing – review and editing. **A. S. Lynnes:** data curation, investigation, methodology, resources, writing – review and editing. **T. I. Morley:** data curation, investigation, methodology, resources, writing – review and editing. **J. Forcada:** data curation, formal analysis, investigation, resources, software, validation, writing – original draft, writing – review and editing.

## Ethics Statement

All procedures performed in this study involving animals were in accordance with permits issued under the Antarctic Treaty Act (Antarctic Act 1994) and approved by the British Antarctic Survey Animal Welfare and Ethics Review Body.

## Conflicts of Interest

The authors declare no conflicts of interest.

## Supporting information


**Figure S1.** Plots of paired principal components (PC) of sea ice variables describing the seasonal sea ice cycle around the South Orkney Islands. See methods section for variable description. The *x‐* and *y*‐axes labels show the % of variance explained by the respective PCs, in parentheses.
**Figure S2.** Coherence wavelet power spectra of pairs of species and sea ice principal components. Cool and warm colours represent areas of correlation between 0 and 1. Regions of significantly high temporal coherence compared to a null model (red noise) are designated with black contours, and paler areas delimit the cone of influence, with outside values being less reliable due to edge effects. Small arrows show phase differences between species *x* and *y*. With right pointing arrows *x* and *y* are in phase; to the left, *x* and *y* are in anti‐phase; pointing up, *y* leads *x* by *π*/2; and pointing down, *x* leads *y* by *π*/2.

Data S1.

## Data Availability

The data that support the findings of this study are openly available from British Antarctic Survey Polar Data Centre at https://doi.org/10.5285/8e910ac8‐6f52‐4548‐bbcb‐78474b559294. The satellite measurements of percent sea ice concentration data were obtained from the NOAA National Centers for Environmental Information (NCEI) at https://doi.org/10.7289/V5CZ3562 and https://doi.org/10.25921/RE9P‐PT57. Our calculated data are openly available from British Antarctic Survey Polar Data Centre at https://doi.org/10.5285/0283c3e4‐2164‐4dfb‐a066‐0c49b816d7b6.
