# Supplementary material for: Temporary Absence of Warming in the Northern Weddell Sea Validates Expected Responses of Antarctic Seals to Sea Ice Change
Source: Glob Chang Biol. 2025 Jun 18;31(6):e70290. doi: 10.1111/gcb.70290 (PMC12175055; doi:10.1111/gcb.70290)
Supplement: Supplementary file 1 — Figure S1. Plots of paired principal components (PC) of sea ice variables describing the seasonal sea ice cycle around the South Orkney Islands. See methods section for variable description. The x‐ and y‐axes labels show the % of variance explained by the respective PCs, in parentheses. Figure S2. Coherence wavelet power spectra of pairs of species and sea ice principal components. Cool and warm colours represent areas of correlation between 0 and 1. Regions of significantly high temporal coherence compared to a null model (red noise) are designated with black contours, and paler areas delimit the cone of influence, with outside values being less reliable due to edge effects. Small arrows show phase differences between species x and y. With right pointing arrows x and y are in phase; to the left, x and y are in anti‐phase; pointing up, y leads x by π/2; and pointing down, x leads y by π/2. [file GCB-31-e70290-s001.pdf]

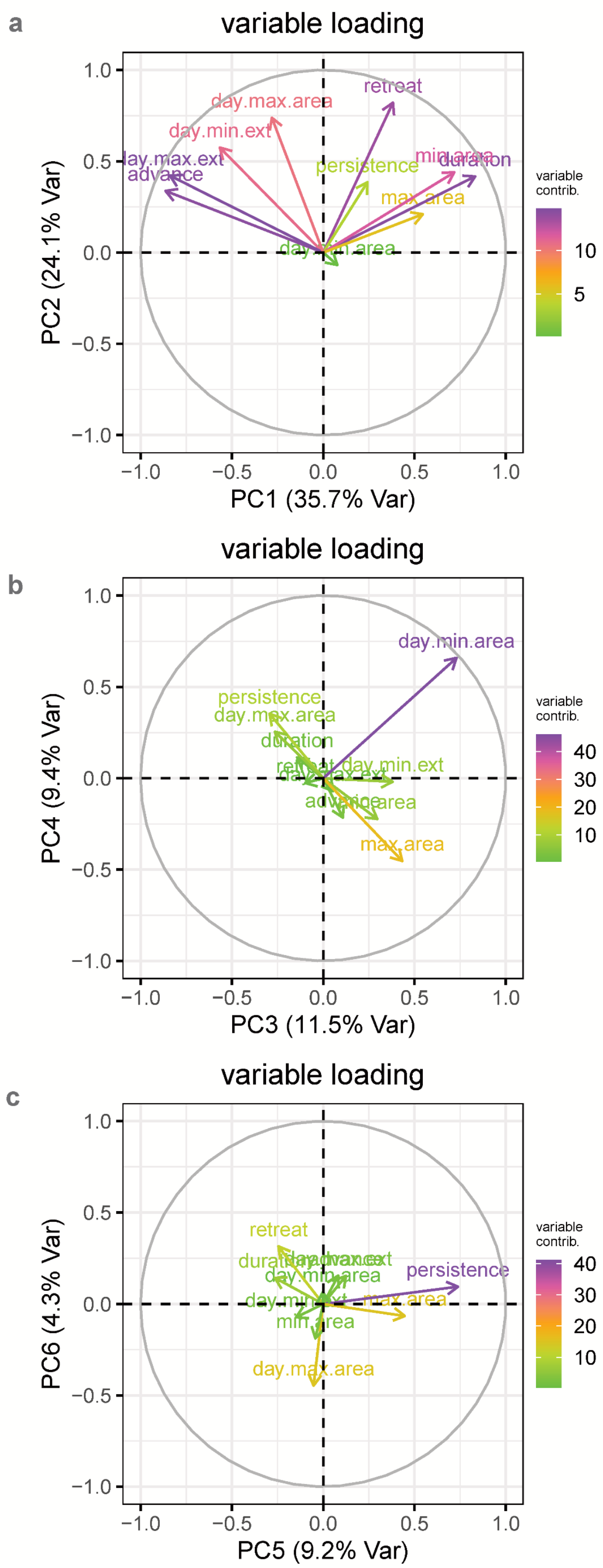

Figure S1. Plots of paired principal components (PC) of sea ice variables describing the seasonal sea ice cycle around the South Orkney Islands. See methods section for variable description. The x and y axes labels show the % of variance explained by the respective PCs, in parentheses.

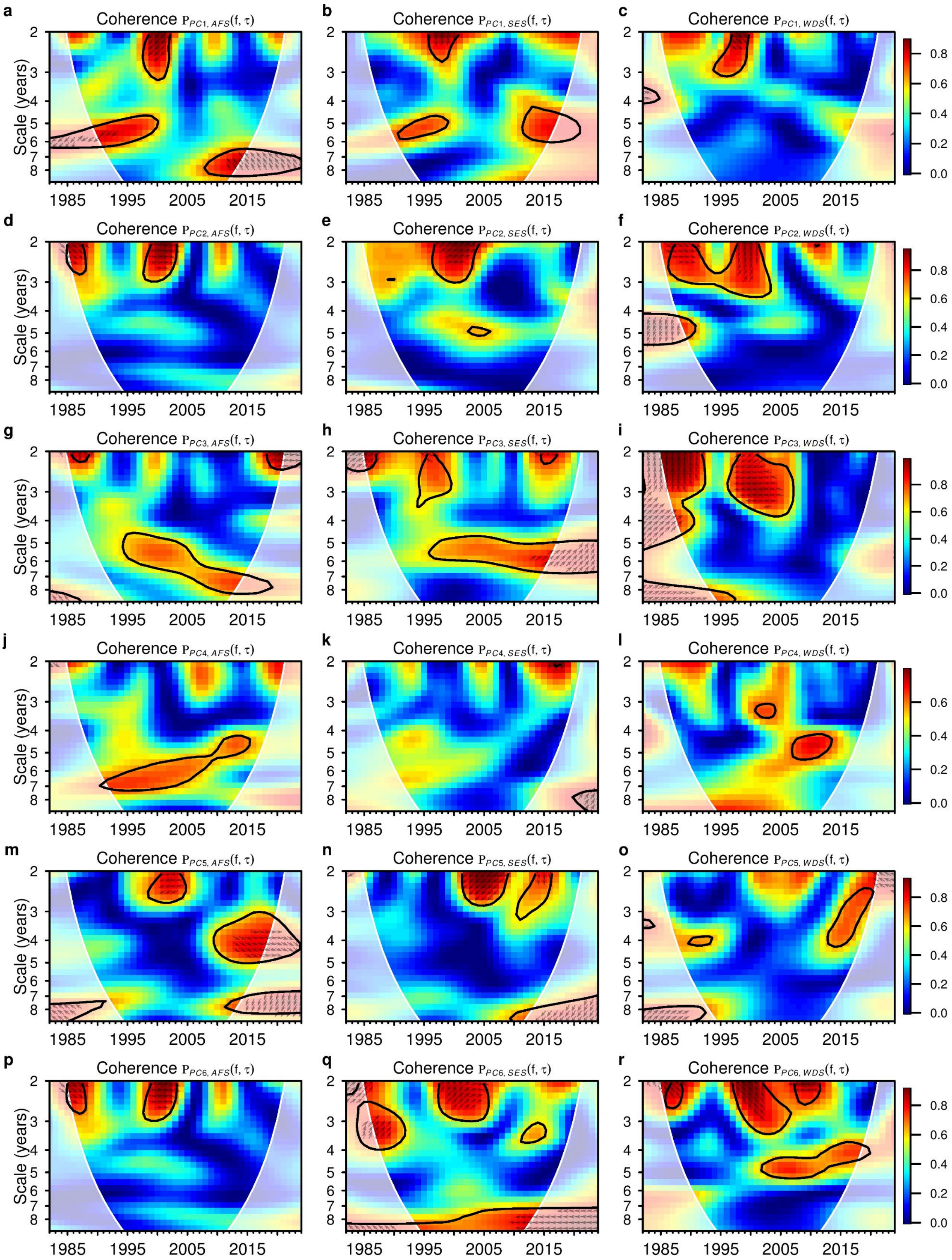

Figure S2. Coherence wavelet power spectra of pairs of species and sea ice principal components. Cool and warm colours represent areas of correlation between 0 and 1. Regions of significantly high temporal coherence compared to a null model (red noise) are designated with black contours, and paler areas delimit the cone of influence, with outside values being less reliable due to edge effects. Small arrows show phase differences between species x and y. With right pointing arrows x and y are in phase; to the left, x and y are in anti-phase; pointing up, y leads x by π/2; and pointing down, x leads y by π/2.
