# Supplementary material for: Temporary Absence of Warming in the Northern Weddell Sea Validates Expected Responses of Antarctic Seals to Sea Ice Change
Source: Glob Chang Biol. 2025 Jun 18;31(6):e70290. doi: 10.1111/gcb.70290 (PMC12175055; doi:10.1111/gcb.70290)
Supplement: Supplementary file 2 — Data S1. [file GCB-31-e70290-s002.pdf]

Table S1. Results of the PCA of sea ice variables: day of advance, day of retreat, ice season duration, sea-ice persistence, day of minimum sea ice extent, day of maximum extent, day of minimum sea ice area, day of maximum area, minimum area covered, and maximum area covered (See main text for definitions). Values under 'loadings' depict R^2 statistics with P-value ≤0.04 for variables most associated with each principal component.

	PC1	PC2	PC3	PC4	PC5	PC6	PC7	PC8	PC9
Variance	3.681	2.394	1.183	0.943	0.852	0.389	0.272	0.209	0.078
%Variance explained	36.81	23.94	11.83	9.43	8.51	3.89	2.71	2.09	0.78
Cumulative %var.	36.81	60.75	72.58	82.00	90.52	94.41	97.13	99.22	100
PC Loadings									
Advance day	-0.857	0.353							
Retreat day	0.414	0.820				0.314			
Duration	0.861	0.372							
Persistence		0.377	-0.310	0.716	0.396				
Day minimum extent	-0.552	0.581	0.410				-0.367		
Day maximum extent	-0.837	0.426							
Day minimum area			0.729	0.556					
Day maximum area		0.722				-0.413			
Minimum area	0.734	0.408						-0.367	
Maximum area	0.584		0.429		0.563				

Table S2. Selection of species interaction parameters ($\nu_{s=r}$, $\nu_{s\neq r}$) in negative binomial population models. Indicator variables *I* with their estimated mean value use subscripts *a* for Antarctic fur seal, *s* for Southern elephant seal, and *w* for Weddell seal. A variable was selected with *I*=1 or deselected when *I*=0, and *p* is the estimated mean model probability based on indicator weights. Ones in a row correspond to effects included in a model, with asterisks identifying parameters with a proportion of more than 95% of the posterior distribution with the same sign as the mean. ΔWAIC is the difference between the WAIC of a model and the lowest WAIC among fitted models. A value between 0 and 2 suggest models with higher support. Only the first ten models are shown.

	$I_{\nu_{aa}}$	$I_{\nu_{sa}}$	$I_{\nu_{wa}}$	$I_{\nu_{as}}$	$I_{\nu_{ss}}$	$I_{\nu_{ws}}$	$I_{\nu_{aw}}$	$I_{\nu_{sw}}$	$I_{\nu_{ww}}$	WAIC-based model selection				
	1	0.04	0.04	0.03	1	0.07	0.04	0.08	0.07	p	Deviance	pWAIC	WAIC	ΔWAIC
1	1*				1*					0.716	1424.7	58.5	1483.2	0
2	1*				1*			1		0.048	1428.9	58.8	1487.7	4.5
3	1*				1*	1				0.043	1424.6	58.6	1483.2	0
4	1*				1*				1	0.041	1426.4	58.8	1485.2	2
5	1*		1		1*				1	0.025	1425.5	58.9	1484.4	1.2
6	1*				1*		1			0.024	1428.3	58.7	1486.9	3.7
7	1*	1			1*					0.023	1424.8	58.7	1486.9	3.7
8	1*			1	1*					0.017	1425.5	58.5	1484.1	0.9
9	1*				1*			1	1	0.006	1431.0	59.0	1490.0	6.8
10	1*				1*	1		1		0.005	1428.6	59.0	1487.5	4.3

Table S3. Indicator-based and *WAIC*-based selection of density-dependence (ν_s) and sea ice fixed effects (γ_s) in single-species negative binomial population models. Indicator variables I with estimated mean weight value underneath, use subscripts *a*, *s*, *w* for species and superscripts PC1 to 6 for synthetic ice variables. Variables were selected with I=1 or deselected when I=0 when model fitting, and column p is the estimated mean probability for a model including combinations of selected PCs, denoted with 1 in the same row. pWAIC is the effective number of parameters, ΔWAIC is the difference between the WAIC of a model and the lowest WAIC among fitted models. Tables show the first 10 models, ranked according to highest *p* estimate.

	I_{ν_a}	$I_{\gamma_a^{PC1}}$	$I_{\gamma_a^{PC2}}$	$I_{\gamma_a^{PC3}}$	$I_{\gamma_a^{PC4}}$	$I_{\gamma_a^{PC5}}$	$I_{\gamma_a^{PC6}}$		WAIC-based model selection			
	0.268	0.033	0.214	0.049	0.054	0.065	0.076	p	deviance	pWAIC	WAIC	ΔWAIC
1	1*							0.166	726.2	21.9	748.1	3.9
2			1*					0.120	738.9	20.4	759.3	15.1
3	1*		1*					0.033	722.8	21.4	744.2	0.0
4						1	1	0.017	738.6	21.1	759.8	15.6
5	1*					1	1	0.015	726.8	22.3	749.1	4.9
6					1			0.013	740.6	20.6	761.2	17.0
7				1				0.010	740.2	20.7	761.0	16.8
8			1*	1				0.009	742.1	20.1	762.4	18.2
9	1*				1			0.008	725.9	22.0	747.9	3.7
10			1*		1			0.008	737.3	21.0	758.3	14.1

	I_{ν_s}	$I_{\gamma_s^{PC1}}$	$I_{\gamma_s^{PC2}}$	$I_{\gamma_s^{PC3}}$	$I_{\gamma_s^{PC4}}$	$I_{\gamma_s^{PC5}}$	$I_{\gamma_s^{PC6}}$		WAIC-based model selection			
	0.857	0.101	0.254	0.183	0.081	0.092	0.178	p	deviance	pWAIC	WAIC	ΔWAIC
1	1*							0.480	498.0	20.1	518.1	8.6
2	1*		1*					0.082	491.5	18.5	509.9	0.4
3	1*			1*				0.065	499.0	20.3	519.3	9.8
4			1*					0.035	491.4	18.1	509.5	0.0
5	1*		1*	1*				0.034	493.0	18.8	511.8	2.3
6	1*					1	1	0.025	497.4	20.1	517.5	8.0
7	1*	1						0.023	503.5	20.9	524.3	14.8

8	1*				1			0.020	498.4	20.2	518.6	9.1
9	1*	1	1*					0.015	497.2	19.4	516.7	7.2
10	1*	1	1	1				0.010	497.2	19.4	516.6	7.1

	I_{ν_w}	$I_{\gamma_w^{PC1}}$	$I_{\gamma_w^{PC2}}$	$I_{\gamma_w^{PC3}}$	$I_{\gamma_w^{PC4}}$	$I_{\gamma_w^{PC5}}$	$I_{\gamma_w^{PC6}}$		WAIC-based model selection			
	0.951	0.080	0.060	0.073	0.103	0.202	0.691	p	deviance	pWAIC	WAIC	ΔWAIC
1	1*							0.579	243.4	16.3	259.7	13.2
2	1*					1	1*	0.128	229.4	18.0	247.4	0.9
3	1*				1			0.045	241.0	17.9	258.9	12.4
4	1*	1						0.037	241.6	17.2	258.8	12.3
5	1*			1				0.032	239.1	18.2	256.3	9.8
6	1*		1					0.024	244.8	16.6	261.4	14.9
7	1*				1	1*	1*	0.021	230.4	18.0	248.3	1.8
8	1*			1		1	1*	0.012	227.9	18.6	246.5	0
9	1*	1				1	1*	0.012	231.2	17.9	249.1	2.6
10	1*		1			1	1*	0.008	230.3	18.6	248.9	2.4
11	1*						1*	<0.001	231.5	17.8	249.3	2.8
12	1*					1		<0.001	247.8	13.9	261.7	15.2

Table S4. *WAIC*-based model selection of multi-species models including density density-dependence (ν_S) and sea ice fixed effects (γ_{PC*}) for Antarctic fur seals (A), Southern elephant seals (S) and Weddell seals (W) in negative binomial population models. All the models include density-dependence, and only the most probable sea ice fixed effects for each species (**Tables S2-4**) are considered. pWAIC is the effective number of parameters.

	Density-dependence			Sea-ice effects									
	ν_A	ν_S	ν_W	γ_a^{PC2}	γ_s^{PC2}	γ_w^{PC2}	γ_w^{PC4}	γ_w^{PC6}	deviance	pWAIC	WAIC	ΔWAIC	Rank
1	ν_A	ν_S	ν_W						1427.9	59.1	1487.0	11.1	9
2	ν_A	ν_S	ν_W	γ_{PC2}^A	γ_{PC2}^S		γ_{PC4}^W		1424.7	58.4	1483.0	7.1	5
3	ν_A	ν_S	ν_W	γ_{PC2}^A	γ_{PC2}^S				1425.1	58.2	1483.3	7.4	6
4	ν_A	ν_S	ν_W	γ_{PC2}^A	γ_{PC2}^S	γ_{PC2}^W			1425.5	58.6	1484.1	8.2	8
5	ν_A	ν_S	ν_W	γ_{PC2}^A	γ_{PC2}^S			γ_{PC6}^W	1420.3	56.5	1476.8	0.9	2
6	ν_A	ν_S	ν_W	$\gamma_{PC2}^A = \gamma_{PC2}^S$				γ_{PC6}^W	1419.7	56.2	1475.9	0	1
7	ν_A	ν_S	ν_W	$\gamma_{PC2}^A = \gamma_{PC2}^S$			γ_{PC4}^W		1424.1	58.1	1482.3	6.4	4
8	ν_A	ν_S	ν_W					γ_{PC6}^W	1423.3	57.4	1480.7	4.8	3
9	ν_A	ν_S	ν_W	$\gamma_{PC2}^A = \gamma_{PC2}^S = \gamma_{PC2}^W$					1425.3	58.2	1483.4	7.5	7

Table S5. Parameter estimates for best multi-species model including sea-ice effects, named *residual variance model*, and for the model without ice effects, or *total variance model*. Values between squared brackets are 95% credible intervals from simulations of posterior distributions. See Methods in main text for parameter definitions.

Parameter	Total variance model	Residual variance model	Sea ice contribution to variance components
r_A	32.4 [11.6,49.3]	32.7 [12.2, 49.2]	
r_S	35.4 [14.6,49.4]	37.6 [17.5, 49.5]	
r_W	24.1 [2.6,48.7]	26.6 [4.2, 48.9]	
$\bar{\alpha}$	2.177 [0.672, 3.733]	2.036 [0.637, 3.411]	
α_A	2.080 [0.459, 3.633]	2.000 [0.510, 3.456]	
α_S	2.310 [0.955, 3.834]	2.137 [0.915, 3.470]	
α_W	2.019 [0.901, 3.043]	1.987 [1.083, 2.857]	
$1-\nu_A$	-0.227 [-0.396, -0.052]	-0.217 [-0.375, -0.057]	
$1-\nu_S$	-0.359 [-0.591, -0.151]	-0.330 [-0.534, -0.141]	
$1-\nu_W$	-0.905 [-1.339, -0.409]	-0.892 [-1.261, -0.494]	
γ_A^{PC2}		-0.103 [-0.182, -0.024]	
γ_S^{PC2}		-0.103 [-0.182, -0.024]	
γ_W^{PC6}		0.662 [0.275, 1.052]	
σ_δ^2	0.081 [0.031, 0.164]	0.075 [0.030, 0.149]	$C_\delta = 0.074$
$\sigma_{\epsilon_A}^2$	0.129 [0.046, 0.257]	0.115 [0.041, 0.232]	$C_{\epsilon_A} = 0.109$
$\sigma_{\epsilon_S}^2$	0.102 [0.039, 0.204]	0.097 [0.040, 0.192]	$C_{\epsilon_S} = 0.049$
$\sigma_{\epsilon_W}^2$	0.423 [0.079, 0.879]	0.306 [0.080, 0.646]	$C_{\epsilon_W} = 0.277$
ICC_A	0.396 [0.148, 0.700]	0.406 [0.160, 0.701]	
ICC_S	0.447 [0.184, 0.734]	0.441 [0.187, 0.718]	
ICC_W	0.187 [0.057, 0.500]	0.221 [0.071, 0.506]	
ICC	0.404 [0.163, 0.671]	0.391 [0.169, 0.645]	
